# Multipurpose Sensor Based on a Polymethacrylate Matrix Nanocomposite with Immobilized Gold Nanoparticles for the Determination of Environmental Pollutants

**DOI:** 10.3390/polym18111375

**Published:** 2026-06-01

**Authors:** Daria E. Kuznetsova, Olga A. Bazhenova, Nataliya A. Gavrilenko, Mikhail A. Gavrilenko, Nadezhda V. Saranchina

**Affiliations:** 1 Department of Analytical Chemistry, Tomsk State University, 634050 Tomsk, Russiadce@mail.tsu.ru (N.A.G.); saranchina@mail.tsu.ru (N.V.S.); 2 Department of Chemical Engineering, Tomsk Polytechnic University, 634050 Tomsk, Russia

**Keywords:** tetracycline, polymethacrylate matrix, solid phase extraction, solid phase spectrophotometry, solid phase fluorimetry, colorimetry

## Abstract

An optical sensor based on a polymethacrylate matrix (PMM) with immobilized gold nanoparticles (Au^0^ NPs) has been developed for the determination of pollutants in environmental samples. The nanoparticles are synthesized by chemical reduction of Au(III) to Au^0^ using sodium borohydride, which yields conglomerates of spherical particles with an absorption maximum at 530 nm. The time stability of the nanocomposite is demonstrated, as well as the ability to control the nanoparticle loading in the matrix by varying the concentration of the HAuCl_4_ solution. The analytical capability of the PMM–Au^0^ system is demonstrated for the direct determination of tetracycline in river water in two linear concentration ranges: 0.001–0.010 mg/L and 0.025–0.100 mg/L, with detection limits of 0.0005 mg/L and 0.012 mg/L, respectively. The determination of tetracycline is based on the enhancement of its intrinsic fluorescence at 520 nm by gold nanoparticles in the solid phase following solid-phase extraction from water in the anionic form H_2_TC^−^ using PMM–Au^0^. The colorimetric determination of thiocyanate anions is based on a color change of the PMM–Au^0^ nanocomposite from red to blue, corresponding to a shift in the plasmon absorption maximum from 530 nm to 630 nm. The sensor exhibits a linear response in the thiocyanate concentration range of 0.3–50.0 mg/L, with a detection limit of 0.1 mg/L. Thus, the multifunctional PMM–Au^0^ sensor has been used for the determination of various analytes employing different modes of analytical signal readout after minimal sample preparation.

## 1. Introduction

Gold nanoparticles (Au^0^ NPs) are particularly attractive for sensor analysis due to their unique optical properties, which can be tuned by changing their size, shape, and surface chemical composition. The key phenomenon—surface plasmon resonance (SPR)—leads to intense light absorption and scattering in the visible region and is widely used in analytical chemistry [1,2,3,4]. Currently, there are two main groups of sensors based on Au^0^ NPs. The first group comprises sensors for surface-enhanced spectroscopy, where analyte interaction with the strong electromagnetic field generated by the nanoparticles is used to enhance the fluorescence signal (surface-enhanced fluorescence), to enhance the infrared signal (surface-enhanced infrared spectroscopy), as well as surface-enhanced Raman spectroscopy [5]. The second group consists of sensors based on monitoring the SPR absorption band, which register color changes or shifts in the SPR band of Au NPs. Such sensors rely on aggregation and disaggregation processes [6,7] and also include nanoparticle surface etching or growth processes [5,8,9].

Despite the successes achieved with colloidal Au^0^ NP systems in optical sensors, their application is limited by poor analytical reproducibility, instability, and short shelf life. Moreover, large amounts of surfactants and stabilizing agents are used, which can affect the reactivity of the nanoparticles, hindering or reducing their applicability in analysis. To overcome these drawbacks, nanoparticle immobilization in a solid phase is employed [10,11]. Among solid supports (glass, polymers, cellulose), polymer matrices are of particular interest because they provide optical transparency, mechanical strength, chemical stability, and the possibility of long-term storage.

Furthermore, polymers enable both spectrophotometric and colorimetric modes of analytical signal readout, including digital image processing using a smartphone [12]. Of special note is the synthesis of gold nanoparticles directly in the solid polymer phase [10], which adheres to green chemistry principles, eliminates the labor-intensive surface functionalization step, and yields stable nanocomposites with controlled Au^0^ NP loading.

Among synthetic polymer materials, poly(methyl methacrylate) (PMM) is of interest. The polymer is optically transparent in the visible and near-UV ranges and possesses the required physicochemical properties for solving many applied problems in analytical chemistry, medicine, and biotechnology [13]. In contrast to colloidal systems, where nanoparticles tend to aggregate during storage and require surfactants for stabilization—which can reduce nanoparticle reactivity, immobilization of Au^0^ NPs into PMM ensures high temporal and chemical stability and opens up possibilities for creating portable analytical devices. It is also worth noting that the extremely low gold content in the matrix (10^−6^ wt%) is critically important for practical application of the proposed sensor. First, this removes the economic constraints typical of analytical systems based on noble metals, and second, it eliminates the inner filter effect (in fluorescence measurements) and provides high sensitivity due to efficient extraction of the target analyte throughout the entire PMM volume, not only its surface layer.

Incorporating Au^0^ NPs into PMM further enhances determination sensitivity, since fluorophores located near the surface of metal nanostructures can exhibit a significant increase in fluorescence quantum yield due to the metal-enhanced fluorescence (MEF) effect [14,15,16,17,18]. This effect arises from local enhancement of the plasmon resonance absorption near the Au^0^ NPs surface. A fundamental requirement for achieving metal-enhanced fluorescence is precise control over the fluorophore–nanoparticle separation distance. As demonstrated in several studies [19], fluorescence enhancement occurs at distances of 5–20 nm, whereas separations below 5 nm are dominated by non-radiative quenching. Consequently, previously reported MEF sensors typically require the incorporation of dielectric spacer layers to prevent fluorescence quenching. In contrast, the rigid PMM matrix intrinsically functions as a built-in spacer, maintaining the optimal distance between the analyte (fluorophore) and the AuNPs exclusively within the enhancement regime, thereby effectively suppressing non-radiative energy transfer.

In addition to classical instrumental methods such as fluorimetry and spectrophotometry, the analytical signal can be recorded with a digital camera, meeting the current demands for accessibility and rapid analysis. In recent years, portable analytical tools that allow measurements directly at the sampling site have been actively developing. Digital colorimetry occupies a key place among them due to the simplicity of the hardware setup and the widespread availability of digital imaging equipment. Smartphones play an important role in miniaturizing such methods; their built-in cameras, combined with image processing software, provide high measurement accuracy. This paves the way for mobile procedures with accuracy comparable to laboratory procedures on stationary equipment [20]. The current trend in using PMM includes minimizing sample preparation by combining solid-phase extraction and digital colorimetry, as well as using a smartphone to process the sensor color image in RGB coordinates and color difference ΔE [21]. The detection limits achieved with digital colorimetry are comparable to those of solid-phase spectrophotometry.

Given this, the aim of this study is to exploit the capabilities and functionality of a sensor based on PMM with immobilized Au^0^ NPs as a promising alternative to currently available colorimetric systems for the determination of environmental pollutants. We have developed a system containing only one type of nanoparticle, opening up prospects for creating cheap, portable, and environmentally friendly analytical devices. Au^0^ NPs are uniformly distributed in the solid polymer phase, remain stable for a long time, use no solvents during preparation and analysis, generate several types of signals (fluorescence, spectrophotometric, and colorimetric), and thus provide much more reliable results.

It is important to emphasize that the majority of reported optical sensors based on gold nanoparticles are designed to operate in a single detection mode either fluorescence or colorimetry and are typically restricted to a narrow range of target analytes. In contrast, we demonstrate that a single PMM-Au^0^ platform can be effectively utilized in two distinct analytical readout modalities. The first modality, metal-enhanced fluorescence, enables ultrasensitive tetracycline detection by exploiting the spectral overlap between the localized surface plasmon resonance of the nanoparticles and the fluorescence emission band of the antibiotic. The use of PMM-immobilized Au^0^ NPs effectively suppresses non-radiative energy transfer and ensures that the extracted tetracycline is maintained at a precise separation distance that resides exclusively within the fluorescence enhancement regime. The second modality, plasmonic colorimetry, relies on a fundamentally different mechanism involving the surface etching of the AuNPs by thiocyanate anions. Notably, most existing colorimetric assays for thiocyanate employing colloidal AuNPs depend on analyte-induced nanoparticle aggregation. However, as previously noted, such colloidal systems frequently suffer from poor long-term stability, require surfactants to prevent premature aggregation, and are highly susceptible to variations in background ionic strength. Implementing Au^0^ NPs immobilized on a PMM substrate successfully overcomes these limitations while significantly minimizing matrix effects from complex real-world samples on the analytical signal. This dual-mode, multi-analyte functionality, achieved with a single and readily fabricated PMM-Au^0^ platform, represents a substantial advancement over conventional single-function sensors and opens promising avenues for the development of portable, cost-effective devices for environmental monitoring.

This work shows that the multipurpose sensor can be used to determine different analytes, e.g., tetracycline in river water by fluorescence or peak area of the anionic form, and thiocyanate in formation water by the colorimetric characteristics of the red-to-blue transition resulting from etching of the Au^0^ NP surface by SCN^−^ anions.

## 2. Materials and Methods

### 2.1. Synthesis of Au^0^ Nanoparticles Embedded in PMM

The polymethacrylate matrix is a transparent colorless polymer material containing carbonyl and carboxyl groups that enable solid-phase extraction of both the reagent and the analyte. For polymerization [22], a reaction mixture consisting of 98% methyl methacrylate monomer and 2% polyethylene glycol PEG 400 was poured into a mold made of non-reactive glass and silicone and placed in a thermostat at 70 °C until conversion was achieved. The resulting polymer mass is a transparent copolymerization product of MMA and PEG 400, which exhibits high potential for solid-phase extraction. In this product, PEG 400 forms hydrophilic chains that ensure penetration of the extracted substance into the polymer. The synthesized PMM plates were 0.5 mm thick. Sensors of the required dimensions 4 × 4 mm were obtained by cutting the original plates into smaller pieces.

Stable Au NPs were formed directly in transparent PMM by reducing Au(III) ions after solid-phase extraction. In the first step, Au(III) was immobilized into the PMM–Au^3+^ matrix from an HAuCl_4_ solution with a concentration of 2.5–25.0 mg/L. For this purpose, 60 PMM plates were stirred with 15 mL of the solution on a rotator for 5 min. In the second step, the PMM–Au^3+^ plates were immersed in a reducing agent solution. A 1% NaBH_4_ solution (reduction time 1 min) and a 5% ascorbic acid solution (reduction time 5 min) were used as reducing agents of different strengths. The PMM–Au^0^ plates were stored at room temperature without any special storage conditions.

### 2.2. Solutions and Reagents

Stock solutions of 0.5 g/L tetracycline (Sigma-Aldrich, St. Louis, MO, USA), 1.0 g/L thiocyanate (Reachem, Moscow, Russia), 20% trichloroacetic acid (PZCM-Vtormet, Moscow, Russia), 1% sodium borohydride (Reachem, Moscow, Russia) and 5% ascorbic acid were prepared by dissolving their weighed samples or aliquots in deionized water obtained using a Millipore Simplicity (Merck Millipore, Burlington, MA, USA) water purification system. To achieve the required pH, 0.1–10.0 M HCl, 0.1 M NaOH, and standard pH-metric buffers (Ekroskhim, Saint Petersburg, Russia) were used. Working solutions of HAuCl_4_ (Acros Organics, Munha Plaza, MD, USA) were prepared on the day of the experiment by diluting a commercial standard with a concentration of 1 g/L. Reagents were used without additional purification.

### 2.3. Experimental Methodology

Solid-phase extraction of tetracycline and thiocyanate into the PMM–Au^0^ volume was carried out in a 5 mL test tube. An aqueous sample solution 10–100 µL together with a solution for achieving the required pH was diluted to 4 cm^3^ with distilled water, and then a PMM–Au^0^ plate was introduced. The test tube contents were stirred for 20 min at temperatures between 20 and 90 °C, and the absorption spectra for thiocyanate or fluorescence spectra for tetracycline were recorded, as well as the color of the obtained sensors was captured using a smartphone after image processing in the RGB color coordinate system.

### 2.4. Instrumentation and Operating Parameters

Absorption spectra, optical densities of polymer matrices and solutions, as well as peak areas were recorded on a Shimadzu UV-1800 spectrophotometer (Shimadzu, Kyoto, Japan) with UVProbe version 2.31 software. The optical characteristics of the matrices after the tetracycline extraction process were measured relative to the original PMM. Fluorescence spectra of the matrices were recorded using an Agilent Cary Eclipse spectrofluorometer (Agilent, Waldbronn, Germany). Fluorescence excitation of tetracycline in PMM–Au^0^ for image scanning was performed using an Ultraviolet analytical cabinet “UVC-HD” (PetroChem, Moscow, Russia). PMM–Au^0^ was additionally examined by SEM on a high-resolution Apreo 2 S field-emission scanning electron microscope (Thermo Fisher Scientific, Waltham, MA, USA) at an accelerating voltage of 10 kV and a current of 0.4 nA. A Multi Bio RS-24 multi-rotator (BioSan, Riga, Latvia) was used for stirring solutions with matrices at 50 rpm, and an MTH-100 thermoshaker (Miulab, Moscow, Russia) was used for stirring with heating. The pH values of the solutions were measured using an I-160 laboratory-grade ion meter (Izmeritelnaya Tekhnika, Korolev, Russia) with silver chloride reference electrodes. A laboratory centrifuge Stegler CM-600C (NV-Lab, Moscow, Russia) was used for centrifugation of real samples during the sample preparation step.

The colored PMM plates after contact with the analyte solution were placed on a sheet of white paper and scanned using a smartphone camera from a distance of 25 cm. The digital images obtained were processed using the ColourGrab mobile application by pointing the cursor at the center of each plate, and their R, G, B color coordinates were determined. The analytical parameters used for the interpretation of the colorimetric data obtained were both the color coordinates (R, G, B) and the values calculated from these coordinates. For both types of determination, the color difference ΔE in the RGB system can be used as an analytical signal, calculated according to the formula:

ΔE = ((R − R_0_)^2^ + (G − G_0_)^2^ + (B − B_0_)^2^)^1/2^

where R, G, B, R_0_, G_0_, B_0_ are the red, green, and blue intensity values of the analytical and blank samples, respectively. An iPhone XR smartphone with a 12 MP camera was used in scanning mode for detection. Using scan mode avoids difficulties associated with lighting and differences in smartphone cameras.

## 3. Results and Discussion

### 3.1. Preparation of the PMM–Au^0^

**Figure 1:**
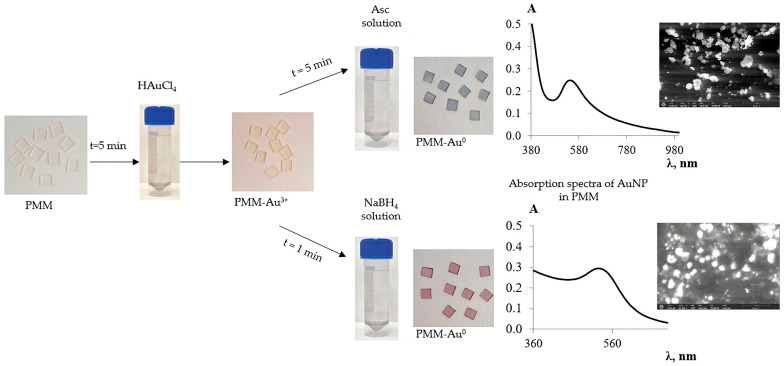
Scheme for the preparation of gold nanoparticles in PMM.

The absorption spectra of the obtained PMM–Au^0^ sensors exhibit an absorption maximum at a wavelength of 535 nm (Figure 1), which corresponds to the synthesis scheme of spherical Au^0^ NPs [23,24]. SEM images show both individual nanoparticles and clusters of nAu^0^ NPs. The results show that the morphology (spherical or branched structures) and optical properties of the resulting gold nanoparticles can be controlled by varying the nature of the reducing agent during their synthesis. The use of the strong reducing agent NaBH_4_ leads to rapid nucleation and the formation of spherical particles, colored red, with an absorption maximum at 530 nm. Mild reduction with ascorbic acid promotes the formation of anisotropic branched structures of a gray-violet color with a bathochromic shift in the plasmon resonance to 580 nm. This is an important factor for two independent detection modes for environmental pollutants: the Metal-Enhanced Fluorescence (MEF) mode for the determination of tetracycline and the colorimetric detection mode based on a plasmon resonance shift for the determination of thiocyanate.

Table S1 presents examples of Au^0^ NP-containing sensors in comparison with the developed PMM–Au^0^, whose synthesis method is characterized by significant simplicity. The reduction of Au^3+^ ions to Au^0^ occurs within 1–5 min at room temperature without the use of additional stabilizers. Furthermore, PMM–Au^0^ remains optically transparent after nanoparticle synthesis, which advantageously distinguishes it from previously proposed sensors and also allows the measurement of both absorption and fluorescence of the target substance in the solid phase of the nanocomposite.

### 3.2. Determination Procedure

**Figure 2:**
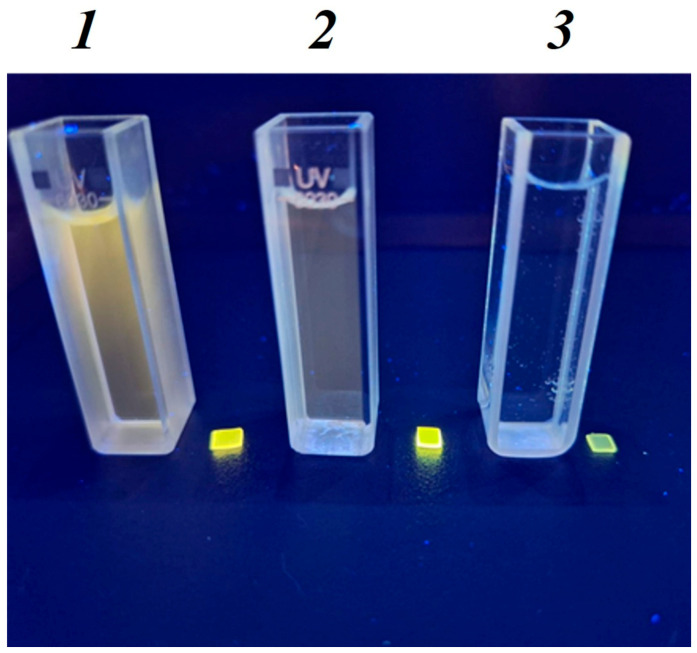
Photograph of luminescence of PMM–Au^0^ sensors and tetracycline solutions (sample volume 4 mL, contact time 20 min), mg/L: 1—11; 2—1.5; 3—0.03.

Tetracycline is an amphoteric molecule with multiple functional groups and can exist in five ionic forms in aqueous solutions depending on pH [25]. It was previously shown that the maximum solid-phase extraction of tetracycline using PMM occurs from solutions with pH from 8.9 to 9.7 in the H_2_TC^−^ form [26]. When using the PMM–Au^0^ nanocomposite, maximum extraction is also achieved in this pH range; however, the distribution coefficient decreases from 588 to 402 mL/g. This effect is associated with partial blocking of the PMM carbonyl groups by gold nanoparticles, which reduces the number of available binding sites for tetracycline. The achieved distribution coefficient value ensures effective solid-phase extraction without suppressing the intrinsic fluorescence of tetracycline and opens up additional analytical possibilities. First, the analyte is concentrated within the sensor volume, increasing the absolute signal (Figure 2). Second, the “rigidity” of the polymer environment restricts the mobility of the tetracycline molecule, suppressing non-radiative processes and thereby increasing the fluorescence quantum yield.

**Figure 3:**
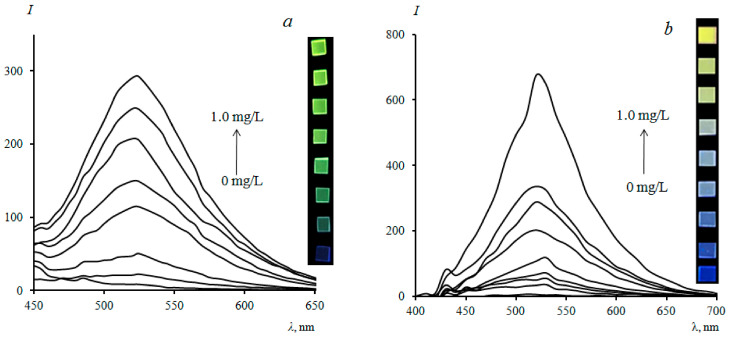
Fluorescence spectra of tetracycline in PMM (**a**) and the corresponding photos of the sensors PMM–Au^0^ (**b**) after contact of the matrix with solutions of different concentrations (excitation at 390 nm) [26].

The fluorescence maximum of tetracycline in PMM is observed at a wavelength of 520 nm (Figure 3a), which coincides with its fluorescence in organic solvents such as acetonitrile and dimethyl sulfoxide [27]. This coincidence proves the similarity of the PMM environment to aprotic solvents, which suppresses intermolecular proton transfer as in the case of aqueous solution. PMM–Au^0^ sensors synthesized using ascorbic acid exhibit intrinsic fluorescence in the blue region of the visible spectrum under UV radiation at 390 nm. The background fluorescence significantly complicates the registration of the tetracycline signal after its extraction. Therefore, for all subsequent studies, PMM–Au^0^ samples synthesized using NaBH_4_ were used, as they did not possess interfering intrinsic fluorescence. Figure 3b shows the fluorescence spectra of tetracycline in PMM–Au^0^ after contact of the matrix with a tetracycline solution.

**Figure 4:**
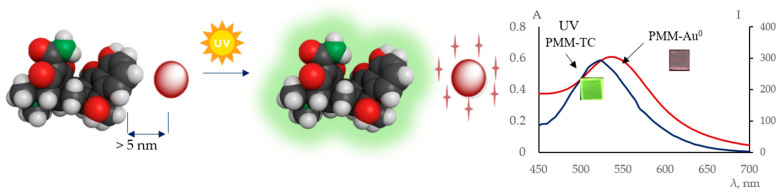
Schematic representation of the metal-enhanced fluorescence effect of tetracycline near the surface of Au^0^ NPs in the solid PMM phase.

The fluorescence intensity of tetracycline naturally increases with increasing concentration, and for the composite PMM–Au^0^ matrix this increase is significantly stronger than for initial PMM, indicating the manifestation of the metal-enhanced fluorescence effect by the nanoparticles. Classical fluorescence quenching of a molecule near the surface of Au^0^ NPs is replaced by enhancement when two conditions are met [23]. First, the distance between the Au^0^ NPs and the fluorophore molecule should be greater than 5 nm, because at shorter distances non-radiative fluorescence quenching occurs. In our system, the polymer matrix acts as a “spacer” and stabilizer, fixing the optimal distance between the Au^0^ NPs and the extracted tetracycline molecules, thus excluding non-radiative energy transfer. Second, spectral overlap between the localized surface plasmon resonance (LSPR) band of the gold nanoparticles and the emission band of the fluorophore is required [28,29]. For spherical gold nanoparticles 10–20 nm in size, the LSPR band lies in the region of 530 nm. In turn, tetracycline has a characteristic fluorescence band with a maximum at 520 nm. The coincidence of these bands (Figure 4) creates resonant conditions under which the local electromagnetic field of the plasmons increases the radiative decay rate of the excited tetracycline molecules [30,31]. As a result, the fluorescence quantum yield of tetracycline increases, leading to enhanced sensitivity of its determination and making possible the direct fluorimetric determination of tetracycline without an elution step, this is an undoubted advantage of the proposed approach.

**Figure 5:**
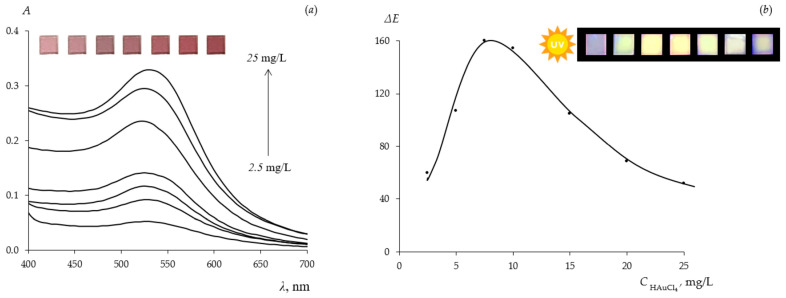
Absorption spectra and the corresponding photos of the sensors PMM–Au^0^ (**a**) and dependence of the analytical signal ΔE on the HAuCl_4_ concentration in the initial solution (**b**) [26].

The effect of the concentration of Au^0^ NPs in PMM on the magnitude of the analytical signal ΔE after tetracycline extraction was investigated (Figure S2). The concentration of Au^0^ NPs in the matrix was varied by varying the concentration of the initial HAuCl_4_ solution in the range from 2.5 to 25.0 mg/L during the immobilization of Au(III) ions. The absorption spectra of the obtained samples (Figure 5a) show an increase in the intensity of the plasmon resonance maximum at 530 nm, associated with the concentration of synthesized Au^0^ NPs in the polymer matrix, corresponding to an increase in the HAuCl_4_ concentration in the initial solution. According to the data in Figure 5b, the analytical signal ΔE reaches a maximum when using HAuCl_4_ solutions with a concentration of 5.0–7.5 mg/L for the synthesis of PMM–Au^0^.

**Figure 6:**
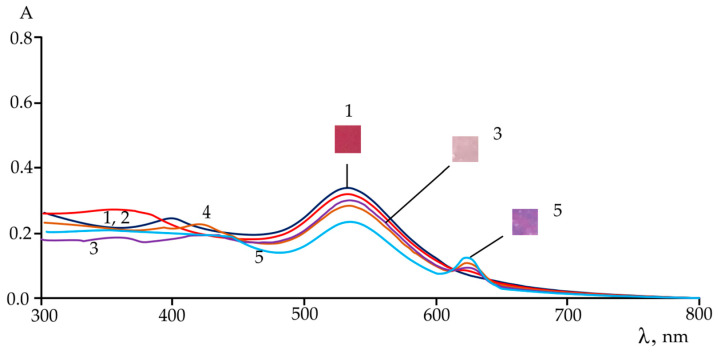
Absorption spectra and the corresponding photos of the sensors PMM–Au^0^ after contact with various concentrations of SCN^−^, mg/L: 1—0; 2—5; 3—15; 4—25; 5—50.

The second target substance for environmental monitoring using the developed PMM–Au^0^ sensor is the thiocyanate anion. In contrast to tetracycline, its determination is based on a color change of the nanocomposite due to a shift in the plasmon absorption from the 530 nm region to the long-wavelength region up to 630 nm, which visually corresponds to a change in the sensor color from red to blue-violet (Figure 6). Thus, in contrast to the fluorescence detection of tetracycline, the determination of thiocyanate is based on direct colorimetric or spectrophotometric measurement of the PMM–Au^0^ sensor color.

The pH of the sample determines the completeness of SCN^−^ extraction through the extraction ability of the PMM–Au^0^ surface and subsequent diffusion into the solid polymer phase [32]. The maximum extraction efficiency is achieved in the pH range 5–7. This is explained by the dependence of extraction and concentration of SCN^−^ in PMM–Au^0^ on the degree of protonation of the matrix surface in an acidic medium. At low pH, the composite surface is protonated with a local positive charge, which promotes electrostatic attraction of the negatively charged SCN^−^ anions. As the pH increases, surface protonation decreases, the charge difference between the carbonyl groups of PMM–Au^0^ and the thiocyanate anions diminishes, and the analytical response of the sensor increases. A further shift of pH into the alkaline region leads to a decrease in the signal, probably due to a shift in the equilibrium of the SCN^−^ accumulation adsorption step on the PMM–Au^0^ surface toward desorption back into the solution.

The spectrophotometric parameter A_630/530_ represents the ratio of absorption coefficients after and before SCN^−^ addition and increases with increasing reaction time, gradually stabilizing after 15 min, indicating completion of the reaction. Therefore, all subsequent experiments were carried out with a reaction time of at least 15 min. The A_630/530_ value gradually increases in the range of 0–35 mg/L, and a further increase in thiocyanate concentration does not lead to significant changes, which exceeds the analytical range of direct spectrophotometric absorption measurement at 630 nm. As the SCN^−^ concentration increases, a gradual increase in A_630_ and a decrease in A_520_ are observed, indicating the incorporation of thiocyanate anions into the coordination shell of the gold nanoparticles [33,34].

**Figure 7:**
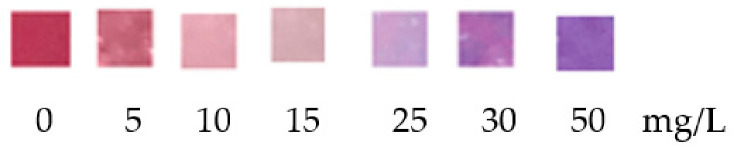
Color scale of PMM–Au^0^ sensors after contact with thiocyanate solutions of different concentrations.

Based on the location of the analytical signal maximum, the conditions for solid-phase extraction of thiocyanate were optimized. The determination error does not exceed 10% at a thiocyanate concentration of up to 27 mg/L, pH 4.8, and a sensor contact time with the sample of 25 min. The color transition contrast in this case significantly exceeds the analytical response upon formation of colored complexes [35,36]. As shown in Figure 7, the sensor color changes from red to violet in the concentration range of 0–50 mg/L, allowing semi-quantitative visual determination of thiocyanate.

**Figure 8:**
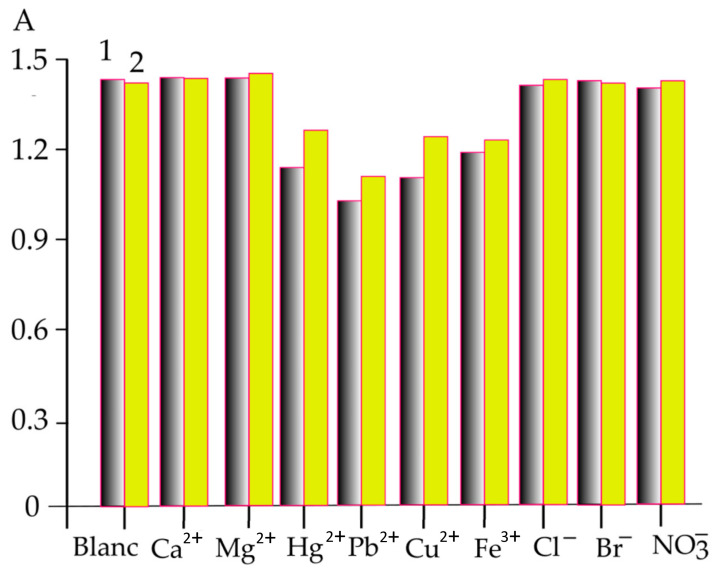
Effect of interfering substances on the determination of SCN^−^ using PMM–Au^0^ and detection: 1—solid-phase spectrophotometry; 2—colorimetry.

The A_630/530_ value in the presence of SCN^−^ was taken as a control value for evaluating the interaction of the sensor with target and background substances. The relative A_630/530_ values in the presence of the most common interfering substances are shown in Figure 8, based on the background composition of formation waters and oilfield waters where thiocyanate anion monitoring is most often required. All anions and hydrocarbons of the water–oil emulsion responded similarly to the standard SCN^−^ concentration of 10 mg/L: the relative A_630/520_ value was about 100% with a deviation within 5%, indicating a negligible influence of these interfering substances.

Most metal cations Mn^2+^, Ag^+^, Ni^2+^, Zn^2+^, Fe^3+^, Cu^2+^, Cd^2+^, Al^3+^, Hg^2+^ interfere with the determination due to complexation with SCN^−^, which reduces the adsorption of thiocyanate on the surface of NPs Au^0^, hinders the formation of the coordination layer, and leads to signal underestimation. However, heavy metal cations are practically absent in formation water [37]. Moreover, adsorption of positively charged metal cations onto the protonated (positively charged) PMM–Au^0^ surface is unlikely under the selected analyte determination conditions in an acidic medium [38]. The obtained results show that most interfering substances from formation water or water–oil emulsion can be separated precisely due to the solid phase of the composite.

### 3.3. Use of PMM–Au^0^ as an Environmental Sensor

**Figure 9:**
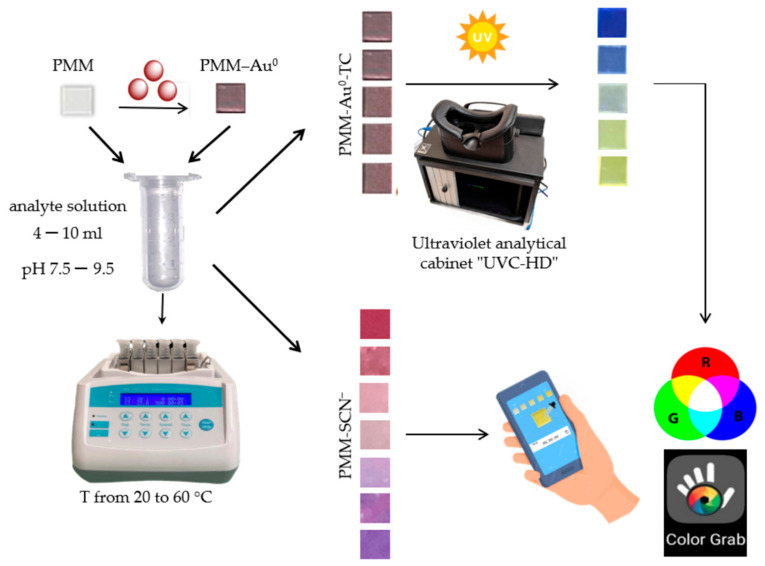
Scheme of the procedure for the determination of tetracycline and thiocyanate using PMM–Au^0^.

The possibility of using the direct fluorescence of tetracycline recorded in PMM–Au^0^ or the color change of the nanocomposite for thiocyanate, combined with digital colorimetry, significantly expands the potential of the proposed determination options. The general analysis scheme (Figure 9) using PMM–Au^0^ is based on calibration dependences and analytical characteristics for the determination of tetracycline and thiocyanate (Table 1).

Based on the conducted studies (Supplementary Materials), procedures for the determination of tetracycline and thiocyanate using PMM–Au^0^ by solid-phase colorimetry were developed and tested on real samples. A detailed description of the procedures is given in Supplementary Materials (Detailed Analytical Procedure).

The accuracy of the analysis was verified by the standard addition method. The results of analyte determination in the analyzed objects are presented in Table 2 and Table 3. The relative standard deviation of the analysis results did not exceed 17%. Quantitative determination of additions of standard solutions of tetracycline and thiocyanate to the analyzed objects is quite accurate.

Thus, the possibility of using PMM–Au^0^ for the direct extraction of substances from the environment followed by colorimetric determination was demonstrated in the concentration range of 0.001–0.1 mg/L with a detection limit of 0.0005 mg/L for tetracycline and 0.3–50.0 mg/L with a detection limit of 0.1 mg/L for thiocyanate at an analyzed sample volume of 4 mL.

## 4. Conclusions

In this work, an optical sensor based on a polymethacrylate matrix with immobilized gold nanoparticles PMM–Au^0^ has been developed for the determination of various environmental pollutants. The synthesis of gold nanoparticles directly in the solid polymer phase eliminates the need for additional stabilizers and adheres to the principles of green chemistry. By selecting the reducing agent NaBH_4_ or ascorbic acid, the nanoparticle morphology can be controlled, yielding either spherical particles with an absorption maximum at 530 nm or branched structures with a maximum at 580 nm. The PMM–Au^0^ nanocomposite was shown to remain stable over time under ambient conditions without any special storage measures, and the gold nanoparticle loading in the matrix can be tuned by varying the concentration of the initial HAuCl_4_ solution, allowing optimization of the analytical signal.

For tetracycline, a metal-enhanced fluorescence mode was realized due to the spectral overlap between the plasmon resonance band of the nanoparticles 530 nm and the emission band of tetracycline 520 nm; the limit of detection for tetracycline in river water is 0.0005 mg/L with a linear range of 0.001–0.100 mg/L. In contrast, the determination of thiocyanate is based on a shift in the plasmon absorption from 530 nm to 630 nm, which visually appears as a color change of the sensor from red to blue; for thiocyanate, a detection limit of 0.1 mg/L was achieved over a concentration range of 0.3–50.0 mg/L. The sensor enables multiple modes of analytical signal readout: fluorimetrically, spectrophotometrically, and colorimetrically using a smartphone for RGB coordinate processing. The effectiveness of the procedures was confirmed by analyzing real samples of river water, formation water, and model water–oil emulsions with good reproducibility (RSD ≤ 17%). Thus, the developed multifunctional PMM–Au^0^ sensor represents a simple, low-cost, and environmentally friendly alternative to existing optical systems for pollutant monitoring after minimal sample preparation.

## Figures and Tables

**Table 1:** Calibration curve equations and analytical characteristics for the determination of tetracycline and thiocyanate using PMM–Au^0^.

Analyte	Calibration Equation	*r*	Linear Range, mg/L	LOD, mg/L
Thiocyanate	ΔE = 10.57 × c	0.983	0.3–25.0	0.1
	B/R = 0.0196 × c	0.993	0.3–50.0	0.2
Tetracycline	ΔE = 10,571 × c	0.992	0.001–0.010	0.0005
	ΔE = 469 × c + 142	0.994	0.025–0.100	0.012

LOD = Limit of Detection.

**Table 2:** Results of tetracycline determination in analyzed objects (*n* = 3–5; *p* = 0.95).

Sample	Added, mg/L	Found, mg/L	RSD, %	Recovery, %
River water	0	<LOD	ND	ND
	0.050	0.053 ± 0.006	8	106
	0.100	0.095 ± 0.012	8	95
Natural water (sample 1)	0	<LOD	ND	ND
	0.010	0.011 ± 0.002	17	112
	0.030	0.029 ± 0.003	7	98
Natural water (sample 2)	0	<LOD	ND	ND
	0.050	0.046 ± 0.006	5	92
	0.100	0.12 ± 0.04	16	116

ND—Not determined, LOD = Limit of Detection, RSD = Relative Standard Deviation.

**Table 3:** Results of thiocyanate determination in oilfield formation water (*n* = 3; *p* = 0.95).

Added, mg/L	Salt Content, g/L	Oil, %	Found, mg/L	RSD, %	Recovery, %
0	10	0	1.22 ± 0.12	4	ND
	30		1.16 ± 0.14	5	ND
	50		1.23 ± 0.25	8	ND
0.50	10	30	1.74 ± 0.16	4	107
	30		1.72 ± 0.21	5	103
	50		1.68 ± 0.24	6	95
1.00	10	50	2.09 ± 0.27	5	89
	30		2.13 ± 0.22	4	93
	50		2.33 ± 0.36	6	113
1.50	10	70	2.68 ± 0.31	5	98
	30		2.79 ± 0.29	4	106
	50		2.80 ± 0.27	4	106

ND—Not determined, RSD = Relative Standard Deviation.

## Data Availability

The original contributions presented in this study are included in the article and Supplementary Material. Further inquiries can be directed to the corresponding author.

## Supplementary Materials

The following supporting information can be downloaded at: https://www.mdpi.com/article/10.3390/polym18111375/s1, Figure S1: SEM image of nanoparticle associates in PMM-Au obtained by reduction with sodium tetraborate (1) and their size distribution (2); Figure S2: The dependence of the color difference ΔE on the concentration of tetracycline for determination using the nanocomposite PMM-Au^0^ (*n* = 5), Table S1: Examples of nanocomposites based on gold nanoparticles prepared by ex situ and in situ methods in the solid phase. Table S2. Comparison of the analytical characteristics of various methods for the determination of thiocyanate and tetracycline. Text S1. Detailed analytical procedure.

## References

[1] Qin L., Zeng G., Lai C., Huang D., Xu P., Zhang C., Cheng M., Liu X., Liu S., Li B. (2018). “Gold rush” in modern science: Fabrication strategies and typical advanced applications of gold nanoparticles in sensing. Coord. Chem. Rev..

[2] Sadiq Z., Safiabadi Tali S.H., Hajimiri H., Al-Kassawneh M., Jahanshahi-Anbuhi S. (2024). Gold nanoparticles-based colorimetric assays for environmental monitoring and food safety evaluation. Crit. Rev. Anal. Chem..

[3] Kumalasari M.R., Alfanaar R., Andreani S. (2024). Gold nanoparticles (AuNPs): A versatile material for biosensor application. Talanta Open.

[4] Yu L., Song Z., Peng J., Yang M., Zhi H., He H. (2020). Progress of gold nanomaterials for colorimetric sensing based on different strategies. TrAC—Trend. Anal. Chem..

[5] Bartolome M., Villasenor M.J., Rios A. (2026). Surface plasmon resonance optical sensors involving nanomaterials as reliable analytical tools: A critical view about performance and applications. Anal. Chim. Acta.

[6] Zhao X., Zhao H., Yan L., Li N., Shi J., Jiang C. (2020). Recent developments in detection using noble metal nanoparticles. Crit. Rev. Anal. Chem..

[7] Polavarapu L., Perez-Juste J., Xu Q.H., Liz-Marzan L.M. (2014). Optical sensing of biological, chemical and ionic species through aggregation of plasmonic nanoparticles. J. Mater. Chem. C.

[8] Wang H., Rao H., Luo M., Xue X., Xue Z., Lu X. (2019). Noble metal nanoparticles growth-based colorimetric strategies: From monocolorimetric to multicolorimetric sensors. Coord. Chem. Rev..

[9] Navarro J., Cepria G., Camacho-Aguayo J., Martín S., Orive A.G., de Marcos S., Galban J. (2024). Towards new fluorometric methodologies based on the in-situ generation of gold nanoclusters. Talanta.

[10] Scroccarello A., Della Pelle F., Del Carlo M., Compagnone D. (2023). Optical plasmonic sensing based on nanomaterials integrated in solid supports. A critical review. Anal. Chim. Acta.

[11] Dykman L.A., Guliy O.I. (2026). Gold-based nanohybrid materials in biosensing. Talanta.

[12] Celikbas E., Guler Celik E., Timur S. (2018). Paper-based analytical methods for smartphone sensing with functional nanoparticles: Bridges from smart surfaces to global health. Anal. Chem..

[13] Saranchina N.V., Bazhenova O.A., Bragina S.K., Semin V.O., Gavrilenko N.A., Volgina T.N., Gavrilenko M.A. (2024). Comparison of methods for the synthesis of silver nanoparticles stabilized in a solid polymethacrylate matrix. Talanta.

[14] Yoon Jeong Y., Kook Y.-M., Lee K., Koh W.-G. (2018). Metal enhanced fluorescence (MEF) for biosensors: General approaches and a review of recent developments. Biosens. Bioelectron..

[15] Oh S.Y., Lee M.J., Heo N.S., Kim S., Oh J.S., Lee Y., Jeon E.J., Moon H., Kim H.S., Park T.J. (2019). Cuvette-type LSPR sensor for highly sensitive detection of melamine in infant formulas. Sensors.

[16] Ferhan A.R., Guo L., Zhou X., Chen P., Hong S., Kim D.H. (2013). Solid-phase colorimetric sensor based on gold nanoparticle-loaded polymer brushes: Lead detection as a case study. Anal. Chem..

[17] Faham S., Khayatian G., Golmohammadi H., Ghavami R. (2018). A paper-based optical probe for chromium by using gold nanoparticles modified with 2,2′-thiodiacetic acid and smartphone camera readout. Microchim. Acta.

[18] Scroccarello A., Della Pelle F., Fratini E., Ferraro G., Scarano S., Palladino P., Compagnone D. (2020). Colorimetric determination of polyphenols via a gold nanoseeds–decorated polydopamine film. Microchim. Acta.

[19] Mendes J.P., dos Santos P.S.S., Dias B., Nunez-Sanchez S., Pastoriza-Santos I., Pérez-Juste J., Pereira C.M., Jorge P.A.S., De Almeida J.M.M.M., Coelho L.C.C. (2024). Exciting surface plasmon resonances on gold thin film-coated optical fibers through nanoparticle light scattering. Adv. Opt. Mater..

[20] Sarkar D.J., Raja R., Kumar V.S., Bhattacharyya S., Pal S., Mukherjee S., Das B.K. (2025). Breaking barrier of binding buffer in colorimetric aptasensing of tetracycline in food fish using peroxidase mimic gold NanoZyme. Methods.

[21] Ponhong K., Nilnit T., Lee C.Y., Kusakunniran W., Saeteard P., Supharoek S.-A. (2025). A facile smartphone-based digital image colorimetric sensor for the determination of tetracyclines in water using natural phenolic compounds induced to grow gold nanoparticles. RSC Adv..

[22] Saranchina N.V., Bazhenova O.A., Bragina S.K., Semin V.O., Gavrilenko N.A., Volgina T.N., Gavrilenko M.A. (2024). Stabilization of gold nanoparticles in a transparent polymer while maintaining the capabilities of a colorimetric glucose sensor. Opt. Mater..

[23] Austin L.A., Kang B., El-Sayed M.A. (2015). Probing molecular cell event dynamics at the single-cell level with targeted plasmonic gold nanoparticles: A review. Nano Today.

[24] Roumyantseva T.B., Dement’eva O.V., Protsenko I.E., Zaitseva A.V., Sukhov V.M., Rudoy V.M. (2019). Plasmonic enhancement of dye fluorescence in polymer/metal nanocomposites. Colloid J..

[25] Jin L., Amaya-Mazo X., Apel M.E., Sankisa S.S., Johnson E., Zbyszynska M.A., Han A. (2007). Ca^2+^ and Mg^2+^ bind tetracycline with distinct stoichiometries and linked deprotonation. Biophys. Chem..

[26] Saranchina N.V., Kuznetsova D.E., Gavrilenko N.A., Gavrilenko M.A. (2025). Solid phase extraction and determination of tetracycline using gold nanoparticles stabilized in a polymethacrylate matrix. Molecules.

[27] Carlotti B., Fuoco D., Elisei F. (2010). Fast and ultrafast spectroscopic investigation of tetracycline derivatives in organic and aqueous media. Phys. Chem. Chem. Phys..

[28] Gartia M.R., Eichorst J.P., Clegg R.M., Liu G.L. (2012). Lifetime imaging of radiative and non-radiative fluorescence decays on nanoplasmonic surface. Appl. Phys. Lett..

[29] Zeng Z., Mizukami S., Fujita K., Kikuchi K. (2015). An enzyme-responsive metal-enhanced near-infrared fluorescence sensor based on functionalized gold nanoparticles. Chem. Sci..

[30] Asadollahi-Baboli M., Mani-Varnosfaderani A. (2014). Rapid and simultaneous determination of tetracycline and cefixime antibiotics by mean of gold nanoparticles-screen printed gold electrode and chemometrics tools. Measurement.

[31] Lee D., Song J., Song G., Pang Y. (2022). Metal-enhanced fluorescence of dyes with quadrupole surface plasmon resonance of silver nanoparticles. Nanoscale Adv..

[32] Saleh D.I., Mahmoud S.F., Etaiw S.E.H. (2022). Ultrasound-assisted synthesis and biological activity of nanosized supramolecular coordination polymers of silver(I) with chloride, thiocyanate, and 4,4′-bipyridine ligands. J. Mol. Struct..

[33] Zhang J., Yang C., Wang X., Yang X. (2012). Colorimetric recognition and sensing of thiocyanate with a gold nanoparticle probe and its application to the determination of thiocyanate in human urine samples. Anal. Bioanal. Chem..

[34] Zhang Z., Zhang J., Qu C., Pan D., Chen Z., Chen L. (2012). Label free colorimetric sensing of thiocyanate based on inducing aggregation of Tween 20-stabilized gold nanoparticles. Analyst.

[35] Zhang Z., Wang H., Chen Z., Wang X., Choo J., Chen L. (2018). Plasmonic colorimetric sensors based on etching and growth of noble metal nanoparticles: Strategies and applications. Biosens. Bioelectron..

[36] Xia J., Dong Z., Cai Y., Guan G., Zhang S., Kovács A., Boothroyd C., Phang I.Y., Liu S., Wu M. (2018). Morphological growth and theoretical understanding of gold and other noble metal nanoplates. Chem.—Eur. J..

[37] Serres-Piole C., Preud’homme H., Moradi-Tehrani N., Allanic C., Jullia H., Lobinski R. (2012). Water tracers in oilfield applications: Guidelines. J. Pet. Sci. Eng..

[38] Gavrilenko N.A., Saranchina N.V. (2010). Solid phase spectrophotometric determination of silver using dithizone immobilized in a polymethacrylate matrix. J. Anal. Chem..

