# Supplementary material for: Multipurpose Sensor Based on a Polymethacrylate Matrix Nanocomposite with Immobilized Gold Nanoparticles for the Determination of Environmental Pollutants
Source: Polymers (Basel). 2026 Jun 1;18(11):1375. doi: 10.3390/polym18111375 (PMC13259052; doi:10.3390/polym18111375)
Supplement: Supplementary file 1 [file polymers-18-01375-s001.zip › polymers-4322885-supplementary.pdf]

Multipurpose sensor based on a polymethacrylate matrix nanocomposite with immobilized gold nanoparticles for the determination of environmental pollutants

Daria E. Kuznetsova, Olga A. Bazhenova, Nataliya A. Gavrilenko, Mikhail A. Gavrilenko, Nadezhda V. Saranchina

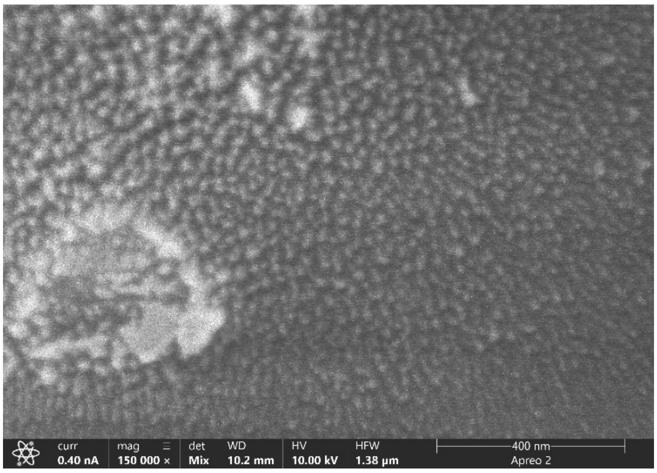

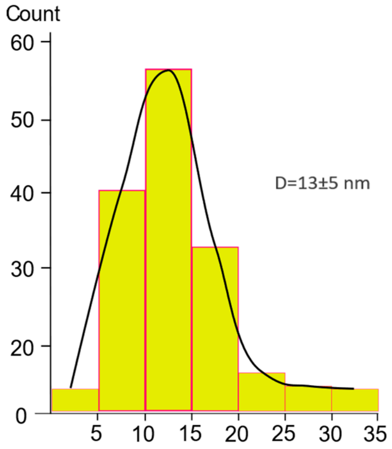

Figure S1. SEM image of nanoparticle associates in PMM-Au obtained by reduction with sodium tetraborate (1) and their size distribution (2)

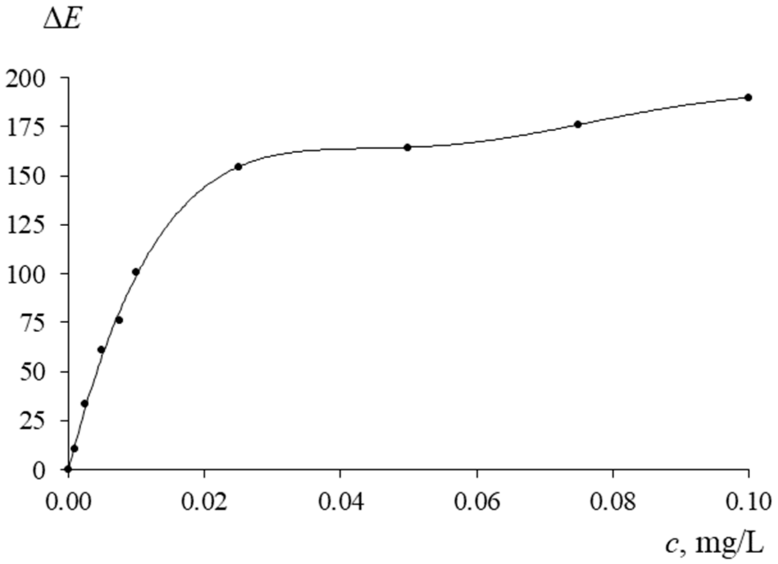

Figure S2. The dependence of the color difference ΔE on the concentration of tetracycline for determination using the nanocomposite PMM-Au^0 (n = 5)

Table S1. Examples of nanocomposites based on gold nanoparticles prepared by ex situ and in situ methods in the solid phase.

Solid phase	Ex / In situ	Nanoparticles	Synthesis method	Analytical signal detection	Application	References
Glass chip	Ex situ	Spherical nanoparticles, 20 nm in size, uniformly distributed on the surface	Labor-intensive synthesis of NPs; use of a large number of reagents	LSPR shift	Melamine	[1]
POEGMA	Ex situ	Spherical nanoparticles, 4 nm in size, embedded in a polymer scaffold	Prolonged NP immobilization process (>12 hours); simplicity of NP deposition onto the substrate	Decrease in LSPR intensity	Pb^{2+}	[2]
Paper	Ex situ	TDA-NP Au have a spherical shape with an approximate nanoparticle diameter of 15 nm	Easy NP Au synthesis; NP modification with TDA takes 2 hours	Change of color of the nanoprobe	Cr^{3+}	[3]
PDA	In situ	NP Au have 2 types of nanoparticles, 20 and 35 nm in size	Long-term modification of PDA with gold nanoparticles over 8 hours	Decrease in LSPR intensity	AOC (Antioxidant Capacity)	[4]
PDMS	In situ	NP Au synthesized in a microchannel have a spherical shape and size distribution of 120–130 nm	Labor-intensive preparation of the polymer chip and long reduction process of NP Au	LSPR shift	Bovine somatotropin (bST)	[5]

Solid phase	Ex / In situ	Nanoparticles	Synthesis method	Analytical signal detection	Application	References
Paper	In situ	Au NPs with citrate groups, 5 nm in size	Simple preparation of paper for Au(I) solution application; rapid incorporation of gold onto paper	Change of color of the nanoprobe	AOC (Antioxidant Capacity)	[6]
DVD/CD	In situ / Ex situ	Plasmonic nanoislands (in situ) 30–50 nm in size or spherical Au NPs 20 nm (ex situ), forming "hot spots"	Repurposing of optical discs; simplicity of NP deposition without complex equipment	Increase in sensitivity to refractive index	Extracellular Vesicles	[7]
MIPs on SPEs	In situ	Hemispherical gold nanoparticles uniformly distributed in the MIP layer	Electrochemical assembly of AuNP and MIP on the SPE surface; process duration 10 minutes	Change in impedance and amperometric current	Glucose	[8]
BNSs (Borophene Nanosheets)	In situ	Highly dispersed spherical Au nanoparticles ~8.5 nm in size, tightly anchored on the nanosheet surface	Use of reducing properties of BNSs; easy synthesis without external reagents	Change of color of the nanoprobe	Glucose	[9]
Snipped human nails	In situ	Nanoparticles of irregular shape (triangles, pentagons, hexagons, and octagons) 80–150 nm in size, forming cluster "hotspots"	"Green" synthesis without reagents; reduction of Au^{3+} by nail collagen over 12 hours at 55 °C	SERS signal enhancement	Malachite green, acid orange	[10]
Au@Pt@Agar	In situ	Spherical Au@Pt nanoparticles uniformly embedded in a 3D hydrogel network	One-step synthesis of hydrogel and NPs; absence of additional purification steps	Change of color of the nanoprobe	Nitrite, glutathione	[11]

Solid phase	Ex / In situ	Nanoparticles	Synthesis method	Analytical signal detection	Application	References
S-rich COF	In situ	Highly dispersed spherical gold nanoparticles uniformly covering the COF surface	Self-reduction of Au^{3+} without an external reducing agent due to Au–S binding; prolonged process of 2 hours	SERS signal enhancement	Letrozole	[12]

References:

1. Oh, S.Y.; Lee, M.J.; Heo, N.S.; Kim, S.; Oh, J.S.; Lee, Y.; et al. Cuvette-type LSPR sensor for highly sensitive detection of melamine in infant formulas. Sensors 2019, 19, 3839.
2. Ferhan, A.R.; Guo, L.; Zhou, X.; Chen, P.; Hong, S.; Kim, D.H. Solid-phase colorimetric sensor based on gold nanoparticle-loaded polymer brushes: lead detection as a case study. Anal. Chem. 2013, 85, 4094–4099.
3. Faham, S.; Khayatian, G.; Golmohammadi, H.; Ghavami, R. A paper-based optical probe for chromium by using gold nanoparticles modified with 2,2′-thiodiacetic acid and smartphone camera readout. Microchim. Acta 2018, 185, 374.
4. Scroccarello, A.; Della Pelle, F.; Fratini, E.; Ferraro, G.; Scarano, S.; Palladino, P.; Compagnone, D. Colorimetric determination of polyphenols via a gold nanoseeds–decorated polydopamine film. Microchim. Acta 2020, 187, 267.
5. SadAbadi, H.; Badilescu, S.; Packirisamy, M.; Wüthrich, R. Integration of gold nanoparticles in PDMS microfluidics for lab-on-a-chip plasmonic biosensing of growth hormones. Biosens. Bioelectron. 2013, 44, 77–84.
6. Choleva, T.G.; Kappi, F.A.; Giokas, D.L.; Vlessidis, A.G. Paper-based assay of antioxidant activity using analyte-mediated on-paper nucleation of gold nanoparticles as colorimetric probes. Anal. Chim. Acta 2015, 860, 61–69.
7. Derin, E.; Yilmaz, E.G.; Erdem, O.; Aslan, Y.; Kafadenk, A.; Çelik, S.; et al. Spatial Designs on Metamaterial Sensors for Enhancing Signals and Detecting Extracellular Vesicles. ACS Appl. Mater. Interfaces 2025, 17, 64048–64064.
8. Cardoso, A.R.A.; Barquinha, P.M.C.; Sales, M.G.F. Enzyme-Free Monitoring of Glucose Using Molecularly Imprinted Polymers and Gold Nanoparticles. Biosensors

2025, 15, 537.

9. Borah, P.; Baruah, D.J.; Mridha, P.; Duarah, R.; Baishya, R.; Das, M.R. In Situ Synthesis of Gold Nanoparticle-Decorated Borophene (Au@BNSs) Nanozymes with Glucose Oxidase and Peroxidase Activity for Colorimetric Detection of Glucose. ACS Omega 2025, 10, 32269–32281.
10. Kumar, J.; Parimi, D.S.; Khan, S.; Panneerselvam, R.; Suresh, A.K. Sustainable synthesis of truncated Au-sensors embedded within robust snipped human nails to monitor dye adulteration in real food samples. Anal. Methods 2025, 17, 4951–4960.
11. Qi, Q.J.; Guan, P.C.; Zhang, Y.X.; Yu, Y.F.; Zhang, G.; Lin, J.S.; et al. In Situ Synthesis of 3D Au@Pt Nanozyme Hydrogel SERS Substrates for Highly Sensitive and Quantitative Sensing. ACS Sens. 2025, 10, 8694–8701.
12. Li, P.; Chen, J.; Xie, Y.; Wu, C.; Zhao, Y.; Luo, X. Constructing high-dispersed Au anchored COFs composites by in-situ self-reduction method for realizing sensitive SERS detection of letrozole. Talanta 2026, 297, 128737.

Table S2. Comparison of the analytical characteristics of various methods for the determination of thiocyanate and tetracycline.

Analyte	Detection Methods	LOD, mg/L	AR, mg/L	References
Thiocyanate	Spectrophotometry	0.1	0.3 – 25.0	This work
		0.02	0.025 –12	[1]
		3	5 – 30	[2]
		0.0215	0.0232–0.0697	[3]
	Colorimetry	0.000174	0.000581 – 4646	[4]
	Potentiometry	0.00555	0.00581 – 5808	[5]
		2.32	0.581– 4005	[6]
Tetracycline	Chromatographic methods	0.001	0.005 – 0.1	[7]
		0.0003	0.001 – 0.500	[8]
	Fluorimetry	0.0004	0 – 0.0444	[9]
		0.0094	0.03 – 1	[10]
		0.0173	0.222 – 35.55	[11]
	Colorimetry	0.015	0.05 – 0.5	[12]
		0.0444	0.222–66.66	[13]
		0.0005<br>0.012	0.001 – 0.010<br>0.025 – 0.100	This work
	ELISA	0.00889	0.0222 – 44.44	[14]

1. Gavrilenko, N. A.; Saranchina, N. V.; Sukhanov, A. V.; Fedan, D. A.; Gavrilenko, M. A. Kinetic determination of thiocyanate by the reaction of bromate with crystal violet immobilized in a polymethacrylate matrix. *J. Anal. Chem.* **2018**, *73(9)*, 894–899.
2. Das, A.; Das, S.; Singh, D. K. Development of Spectrophotometric Method for the Determination of Thiocyanate in Alkaline Medium. *J. Indian Chem. Soc.* **2025**, 102268.
3 Zhao, Y.; Liu, R.; Cui, X.; Fu, Q.; Yu, M.; Fei, Q.; Huan, Y. Colorimetric sensor for thiocyanate based on anti-aggregation of gold nanoparticles in the presence of 2-aminopyridine. *Anal. Sci.* **2020**, *36(10)*, 1165–1169.
4. Cui, X.; Wei, T.; Hao, M.; Qi, Q.; Wang, H.; Dai, Z. Highly sensitive and selective colorimetric sensor for thiocyanate based on electrochemical oxidation-assisted complexation reaction with gold nanostars etching. *J. Hazard. Mater.* **2020**, *391*, 122217.
5. Zainal, S. N.; Rezayi, M.; Alias, Y.; Mahmud, H. E. Self-plasticizing membrane based on Co (II)-porphyrin modified with silver nanoparticles for thiocyanate detection. *Sens. Mater.* **2019**, *31(8)*, 2619–2635.
6. Huy, D. M.; Popova, I. Fast and Simple Approach for Thiocyanate Analysis in Soils. *Commun. Soil Sci. Plant Anal.* **2025**, *56(20)*, 2893–2905.
7. Wang, H. Simultaneous analysis of trace tetracyclines antibiotics in wastewater with ultra-high performance liquid chromatography coupled with mass spectrometry. *J. Pharm. Res. Int.* **2023**, *35(27)*, 15–23.
8. García-Criado, N.; Martín, J.; Santos, J. L.; Aparicio, I.; Alonso, E. Matrix solid-phase dispersion, combined with online SPE-LC-MS/MS, for the determination of tetracyclines and their main transformation products in sludge and agricultural soils. *Anal. Chim. Acta* **2025**, 344749.
9. Che, H.; Nie, Y.; Tian, X.; Li, Y. New method for morphological identification and simultaneous quantification of multiple tetracyclines by a white fluorescent probe. *J. Hazard. Mater.* **2023**, *441*, 129956.
10. Vargas-Muñoz, M. A.; Boudenne, J. L.; Coulomb, B.; Robert-Peillard, F.; Palacio, E. Automated method for the solid phase extraction of tetracyclines in wastewater followed by fluorimetric determination. *Talanta* **2024**, *270*, 125544.

11. Yao, R.; Li, Z.; Liu, G.; Fan, C.; Pu, S. Luminol-Eu-based ratiometric fluorescence probe for highly selective and visual determination of tetracycline. *Talanta* **2021**, *234*, 122612.
12. Ponhong, K.; Nilnit, T.; Lee, C. Y.; Kusakunniran, W.; Saetear, P.; Supharoek, S. A. A facile smartphone-based digital image colorimetric sensor for the determination of tetracyclines in water using natural phenolic compounds induced to grow gold nanoparticles. *RSC Adv.* **2025**, *15(11)*, 8411–8419.
13. Chen, Y.; Xia, Y.; Liu, Y.; Tang, Y.; Zhao, F.; Zeng, B. Colorimetric and electrochemical detection platforms for tetracycline based on surface molecularly imprinted polyionic liquid on Mn_3O_4 nanozyme. *Biosens. Bioelectron.* **2022**, *216*, 114650.
14. Bi, H.; Zhang, C.; Zhao, C.; Zhang, X.; Li, C.; Wang, S.; Shen, Z. A reverse ELISA based on quantum dot-labeled TetR for rapid detection of tetracycline. *Microchim. Acta* **2025**, *192(9)*, 588.

Text S1. Detailed analytical procedure

The developed procedure for tetracycline determination was tested on real river water samples; no preliminary sample preparation of the analyzed samples was required. The colorimetric method for thiocyanate determination using the PMMA–Au^0 sensor was validated on formation water samples with varying anion concentrations, as well as on model water–oil emulsion samples containing 30–70% oil. The formation water samples had salt contents of 10–50 g/dm^3.

The samples were analyzed for tetracycline and thiocyanate content using the standard solution method and calibration curves constructed by the standard addition method. For analysis by the standard solution method, an aliquot portion (0.5–3.6 mL) of the sample was placed into a 5 mL test tube. Then, 0.4 mL of borate buffer (for tetracycline determination) or 0.4 mL of acetate buffer (for thiocyanate determination) was added, the mixture was diluted to 4 mL with distilled water, and a PMMA–Au^0 plate was introduced. The contents of the test tube were stirred for 60 min at 60 °C (for tetracycline determination) or for 25 min at 25 °C (for thiocyanate determination). The plates were then removed from the test tube, and the analytical signal was measured.

For tetracycline determination, the plates were irradiated with light at a wavelength of 365 nm to excite fluorescence. The fluorescence emission in the yellow-green region (520 ± 5 nm) was recorded using a smartphone or an ultraviolet analytical cabinet (UVC-HD). For thiocyanate determination, the color change of the sensor was recorded under normal lighting (red-to-blue colour transition).

When determining analytes by calibration curves constructed using the standard addition method, the procedure described above was followed, with the following modification: reference sample solutions were additionally prepared containing 0.01–0.30 mL of a tetracycline working solution (1.0 mg/L) and 0.02–0.80 mL of a thiocyanate working solution (100 mg/L). Subsequently, a plot of the analytical signal versus the added concentration was constructed, and the resulting straight line was extrapolated to its intersection with the concentration axis.
